# Genome-wide characterization of copy number variations in the host genome in genetic resistance to Marek’s disease using next generation sequencing

**DOI:** 10.1186/s12863-020-00884-w

**Published:** 2020-07-16

**Authors:** Hao Bai, Yanghua He, Yi Ding, Qin Chu, Ling Lian, Eliyahu M. Heifetz, Ning Yang, Hans H. Cheng, Huanmin Zhang, Jilan Chen, Jiuzhou Song

**Affiliations:** 1 Joint International Research Laboratory of Agriculture and Agri-Product Safety, the Ministry of Education of China, Institutes of Agricultural Science and Technology Development, Yangzhou University, Yangzhou, 225009 China; 2 Department of Animal & Avian Sciences, University of Maryland, College Park, MD 20742 USA; 3 Key Laboratory of Animal (Poultry) Genetics Breeding and Reproduction, Ministry of Agriculture, Institute of Animal Science, Chinese Academy of Agricultural Sciences, Beijing, 100193 China; 4 Department of Human Nutrition, Food and Animal Sciences, University of Hawaii at Manoa, Honolulu, HI 96822 USA; 5 Institute of Animal Husbandry and Veterinary Medicine, Beijing Academy of Agriculture and Forestry Sciences, Beijing, 100097 China; 6 Department of Animal Genetics and Breeding, College of Animal Science and Technology, China Agricultural University, Beijing, 100193 China; 7 Faculty of Health Sciences, Jerusalem College of Technology, 9116001 Jerusalem, Israel; 8 USDA, Agricultural Research Service, Avian Disease and Oncology Laboratory, East Lansing, MI 48823 USA

**Keywords:** Copy number variation, Chicken, Inbred lines, Recombinant congenic strains, Marek’s disease, Next generation sequencing

## Abstract

**Background:**

Marek’s disease (MD) is a highly neoplastic disease primarily affecting chickens, and remains as a chronic infectious disease that threatens the poultry industry. Copy number variation (CNV) has been examined in many species and is recognized as a major source of genetic variation that directly contributes to phenotypic variation such as resistance to infectious diseases. Two highly inbred chicken lines, 6_3_ (MD-resistant) and 7_2_ (MD-susceptible), as well as their F_1_ generation and six recombinant congenic strains (RCSs) with varied susceptibility to MD, are considered as ideal models to identify the complex mechanisms of genetic and molecular resistance to MD.

**Results:**

In the present study, to unravel the potential genetic mechanisms underlying resistance to MD, we performed a genome-wide CNV detection using next generation sequencing on the inbred chicken lines with the assistance of CNVnator. As a result, a total of 1649 CNV regions (CNVRs) were successfully identified after merging all the nine datasets, of which 90 CNVRs were overlapped across all the chicken lines. Within these shared regions, 1360 harbored genes were identified. In addition, 55 and 44 CNVRs with 62 and 57 harbored genes were specifically identified in line 6_3_ and 7_2_, respectively. Bioinformatics analysis showed that the nearby genes were significantly enriched in 36 GO terms and 6 KEGG pathways including JAK/STAT signaling pathway. Ten CNVRs (nine deletions and one duplication) involved in 10 disease-related genes were selected for validation by using quantitative real-time PCR (qPCR), all of which were successfully confirmed. Finally, qPCR was also used to validate two deletion events in line 7_2_ that were definitely normal in line 6_3_. One high-confidence gene, *IRF2* was identified as the most promising candidate gene underlying resistance and susceptibility to MD in view of its function and overlaps with data from previous study.

**Conclusions:**

Our findings provide valuable insights for understanding the genetic mechanism of resistance to MD and the identified gene and pathway could be considered as the subject of further functional characterization.

## Background

Marek’s disease (MD) is a lymphoproliferative disease of chickens caused by a highly oncogenic *Gallid alphaherpesvirus* 2, a naturally occurring alphaherpesvirus [1], which goes through a complex life cycle of four main phases [2, 3]: an early cytolytic phase at 2–7 days post infection (dpi), a latent phase around 7–10 dpi, a late cytolytic phase and finally, a proliferation phase after 28 dpi. MD currently remains a neoplastic disease of chickens of serious concerns to the global poultry industry. The control of MD mainly relies on vaccination; however, the vaccination efficacy is being reduced due to newly emerging strains of Marek’s disease virus (MDV) with escalated virulence. Enhancing genetic resistance to MD in poultry is an important long-term goal to better control MD. Therefore, understanding of genetic basis of resistance to MD and improving MD resistance in chickens are of great interest for the poultry industry and animal welfare. In order to optimally implement this control strategy through marker assisted selection (MAS) and to understand the etiology and mechanisms of MD, it is necessary to identify more specific genes and variants with respect to MD resistance.

Genetic variations play crucial roles in phenotypic diversity [4], some of which may underlie major mechanisms that account for variations in disease resistance. The identification of structural variations and potential genetic markers is very important for better understanding of disease resistance, as well as genomic prediction and genetic improvement by selection. There are several types of genetic variations, where copy number variation (CNV) is one of the important types. According to current knowledge, CNV is a type of important genomic structural variation which includes intermediately sized DNA segments that have undergone submicroscopic insertion, deletion, segmental duplication, and complex changes of greater than 1 kilobases (Kb) to several megabases (Mb) in size [5]. It is also a major source of genetic variation underlying phenotypic diversity [6]. Following the first two genome-wide scans for CNVs in human genome [7, 8], a large number of CNV detection studies have been performed, which revealed that CNVs are ubiquitously distributed in genome and can influence the phenotype via regulations of gene expression and gene dosage [9–11]. Besides, numerous studies in other species have also shown that CNVs contributed to phenotypic variation of complex diseases and traits [12–19], including MD in chicken [20–22]. Two major traditional platforms employed in CNV detection are based on SNP chips, one is known as comparative genomic hybridization (CGH) array, and the other is SNP genotyping array. However, due to the limitation in resolution and sensitivity, it is difficult to exhaust small CNVs shorter than 10 kb in length in detection and to identify the precise boundaries of CNVs. In recent years, a variety of CNV detection approaches based on next-generation sequencing (NGS) were proposed, which offer promising alternatives as they have higher effective resolution and sensitivity to discover CNVs with more types and wider size ranges. Advances in NGS have enhanced the new platform for more detailed characterization of CNVs in genomes [23–25].

The primary aim of the present study is to perform a genome-wide CNV analysis by next generation sequencing in the effort to identify CNVs that may confer the difference in genetic resistance to MD between genetic lines. We applied deep sequencing on samples from nine different genetic chicken lines that significantly vary in genetic resistance to MD, including two chicken inbred lines, line 6_3_ and line 7_2_, their F1 reciprocal cross, and the recombinant congenic strains (RCS). RCSs were developed using line 6_3_ as the progenitor background line and line 7_2_ as the progenitor donor line. Eventually, a series of 19 RCSs were generated and each contains a random sample of 87.5% line 6_3_ and 12.5% line 7_2_ genome. All of the chicken lines shared a common major histocompatibility complex B*2 haplotype, but the MD resistance/susceptibility differs among the RCS [1]. Furthermore, CNVnator [26] was employed to generate a comprehensive map of CNVRs and genes. qPCR was used to validate the detected CNVRs. Our finding provides valuable insights for understanding the genetic mechanism of resistance to MD, and the identified genes and pathways could greatly facilitate further functional characterization studies.

## Results

### Mapping statistics and CNV detection

**Fig. 1:**
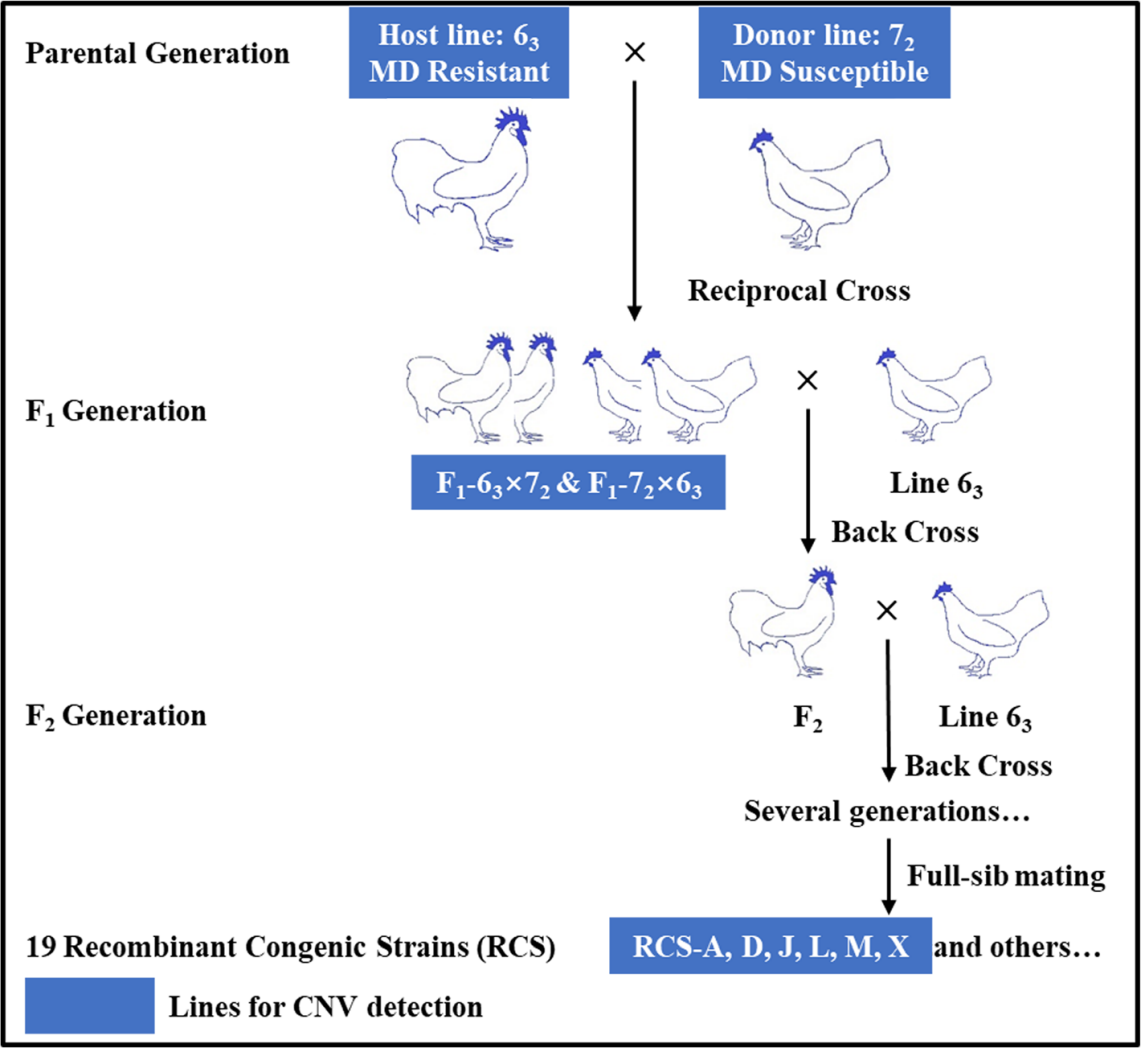
The chicken population used in this study. Chicken lines labeled blue were selected for CNV detection

In this study, we performed whole genome sequencing in nine different chicken lines (Fig. 1) using Illumina paired-end libraries of 26 female chickens. The average numbers of raw reads were approximately 242.26, 230.56, 127.06, 31.39, 34.50, 42.12, 61.06, 57.68 and 37.72 million for lines 6_3_, 7_2_, F_1_, and RCS-A, D, J, L, M, X, respectively (Additional file 1: Table S1). After quality control, an average of 30.42 to 226.30 million reads of each chicken line were successfully aligned to the reference genome (galGal4) with the mapping levels ranging from 90.04 to 98.10% for all the individuals. The sequencing effective depth varied from an average of 5.95× for six RCSs to an average of 19.84× for lines 6_3_, 7_2_ and their F_1_ hybrid, and the average coverage with respect to the reference genome was 88.25%. These high quality alignments provide confident for the subsequent analysis with a minimum of false positives.

**Fig. 2:**
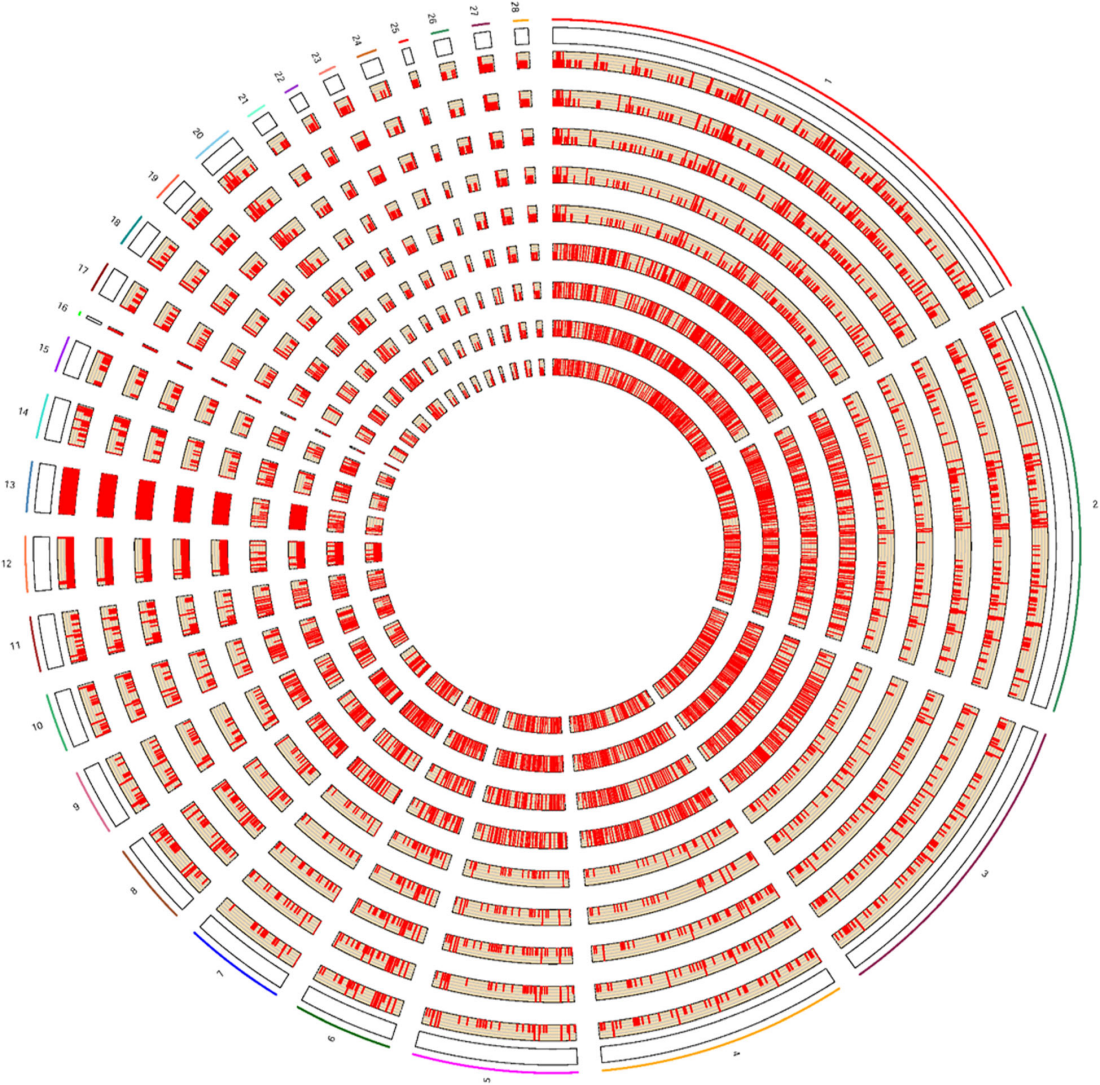
Circos plot illustrating CNV regions in nine chicken lines. Regions with copy number events are plotted within the nine light yellow inner tracks. Copy number changes indicated by two different statuses (deletion or duplication) are shown in the inner circle plot using the RCircos.Histogram.Plot function in RCircos package. The outermost circle displays the chicken chromosomes (chrZ and chrW were excluded). The circles from outside to inside represent Lines 7_2_, 6_3_, F_1_, RCS-A, M, J, D, L and X

**Fig. 3:**
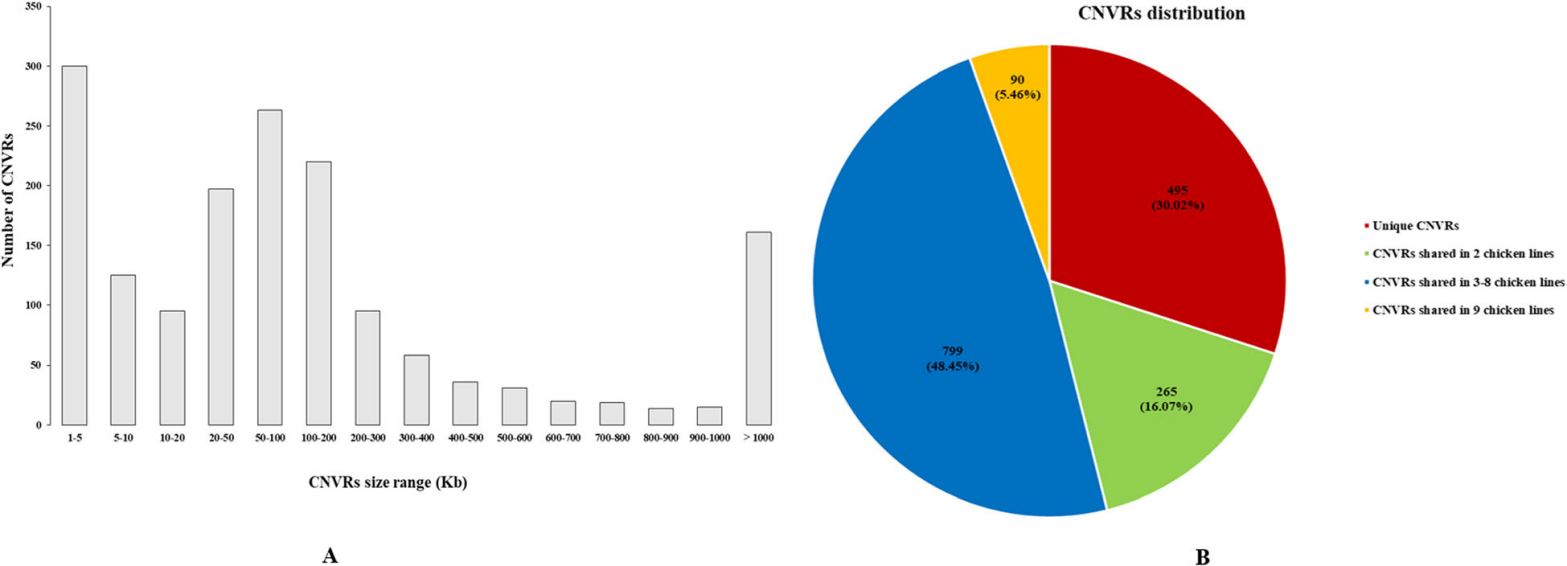
Distribution of CNVRs in nine chicken lines. **a** Length distribution of CNVRs. 1200 (72.8%) CNVR events are shorter than 200 kb. **b** Frequency distribution of unique and overlapped CNVRs. 495 (30.02%) CNVRs occur in only one chicken line and 90 (5.46%) CNVRs are shared in all the nine chicken lines

We then applied the CNVnator software for CNV detection and the average number of CNVs per line, that passed our stringent filtering criteria, was 1888, ranging from 1368 in line RCS-A to 2476 in line RCS-J. The size of these CNVs ranges from 1 Kb to 9.56 Mb, with an average of 95.56 Kb. Detailed statistics of CNV calls are shown in Additional file 2: Table S2. A total of 1649 CNV regions (CNVRs) (Table 1) allowing for CNV overlaps of 1 bp or greater were obtained across all the chicken lines after merging, covering autosomes 1–28, and sex chromosomes Z and W. The chicken CNV map across the genome is shown in Fig. 2. The length of CNVRs ranged from 1 Kb to 18.19 Mb with an average of 0.36 Mb. In total, 1200 (72.8%) out of all CNVRs had sizes varying from 1 to 200 Kb (Fig. 3a). The count of CNVRs on each chromosome was directly proportional to the chromosome length, and five macrochromosomes (chr1–5) possessed a large proportion (874, 53.0%) of all putative CNVRs. The number of CNVRs in different chicken lines varied greatly, ranging from 536 in RCS-L to 852 in RCS-A. Among all CNVRs, 495 (30.02%) were present in only one chicken line and 90 (5.46%) CNVRs are shared in all the nine chicken lines (Fig. 3b). In addition, the CNVRs belonging to gain and loss account for 47.1% (776 events) and 52.9% (873 events), respectively.

**Table 1:** Summary statistics of line-specific and shared CNVRs

Group	No. of CNVRs	Gain	Loss	Total	Common	Harbored genes	Line-specific	Gain	Loss	Harbored genes
6_3_	1193	210	983	1649	90	1360	55	4	51	62
7_2_	1134	205	929				44	7	37	57
F_1_	1111	212	899				82	4	78	135
RCS_A	722	140	582				15	1	14	41
RCS_D	801	555	246				14	7	7	33
RCS_J	949	601	348				72	23	49	300
RCS_L	1031	812	219				190	185	5	559
RCS_M	765	133	632				18	1	17	44
RCS_X	895	617	278				5	2	3	15

### Gene annotation and functional analysis

**Fig. 4:**
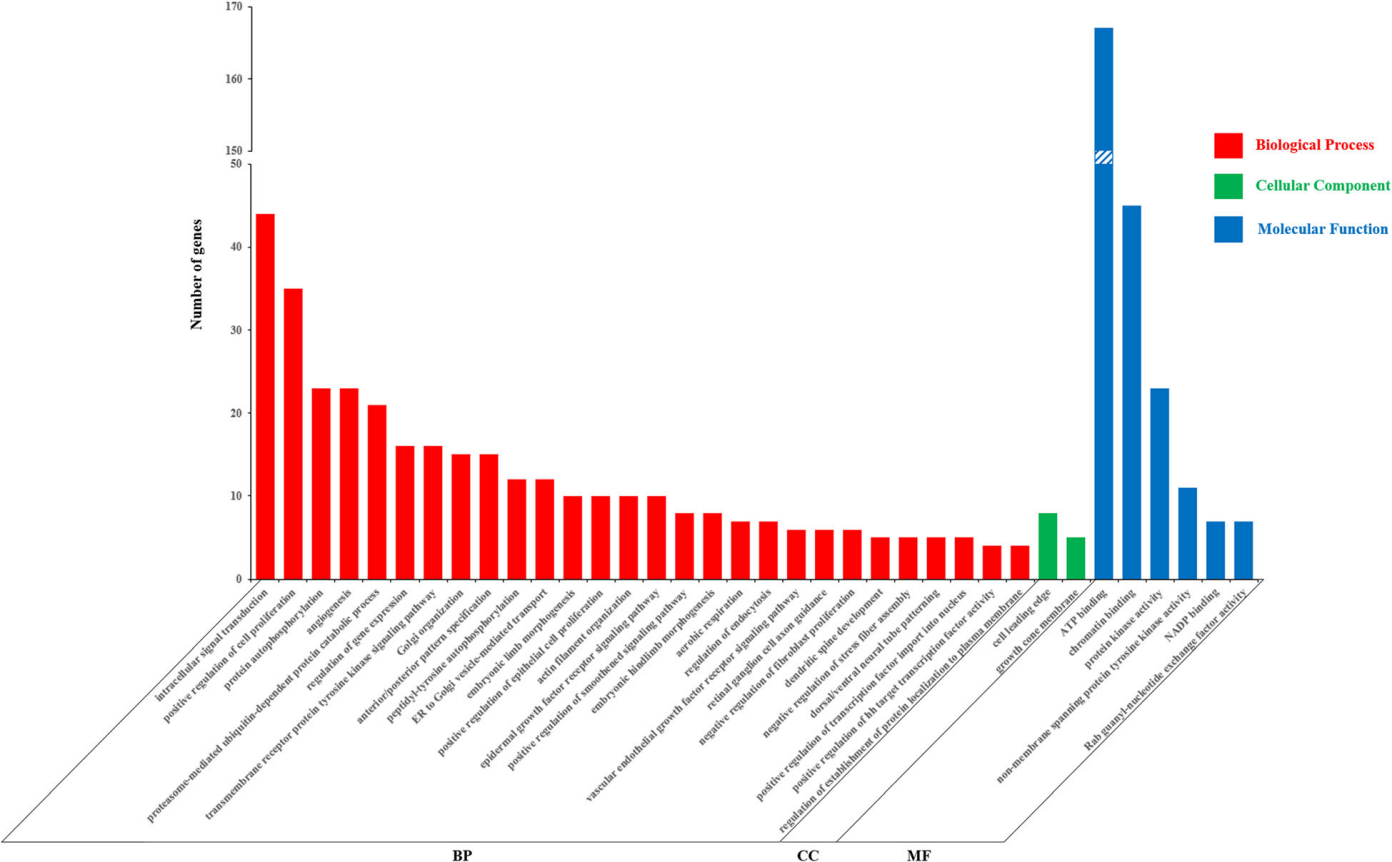
Histogram presentation of Gene Ontology (GO) classification. The y-axis indicates the number of genes in a category, and the x-axis indicates the three main categories: biological process (BP), cellular component (CC) and molecular function (MF). All processes listed had enrichment *P* values < 0.05

The genes harbored in the inferred CNVRs were extracted using custom Perl scripts. As a result, a total of 2588 RefSeq genes within the regions of the 1649 CNVRs were obtained, where a majority of these genes were involved in immunity, tumors and diseases. The identified genes were submitted to DAVID (version 6.8) for GO and pathway enrichment analyses. Using functional annotation clustering, at the highest classification stringency, 145 clusters were formed, where only 9 clusters were chosen after using an enrichment cutoff of > 1.0 (Additional file 3: Table S3). GO terms and KEGG pathways analyses invoked in DAVID yielded 36 significant enriched functional terms (28 terms of biological process, 2 terms of cellular component, and 6 terms of molecular function; *P* < 0.05, Fig. 4). In addition, it yielded 6 significant enriched pathways (*P* < 0.05, Table 2), including the JAK/STAT signaling pathway (gga04630, Additional file 4: Figure S1). The detailed information of all the GO terms and pathways are shown in Additional file 5: Table S4.

**Table 2:** Enriched KEGG pathways of the genes harbored in the CNVRs (*P* < 0.05)

Category	Term	Count	%	***P***-Value
KEGG_PATHWAY	gga00900:Terpenoid backbone biosynthesis	10	0.45	2.31E-03
KEGG_PATHWAY	gga04144:Endocytosis	49	2.22	1.92E-02
KEGG_PATHWAY	gga04146:Peroxisome	20	0.90	2.10E-02
KEGG_PATHWAY	gga04120:Ubiquitin mediated proteolysis	28	1.27	3.06E-02
KEGG_PATHWAY	gga04630:JAK/STAT signaling pathway	25	1.13	4.65E-02
KEGG_PATHWAY	gga04520:Adherens junction	17	0.77	4.85E-02

### PCA analysis and cluster

**Fig. 5:**
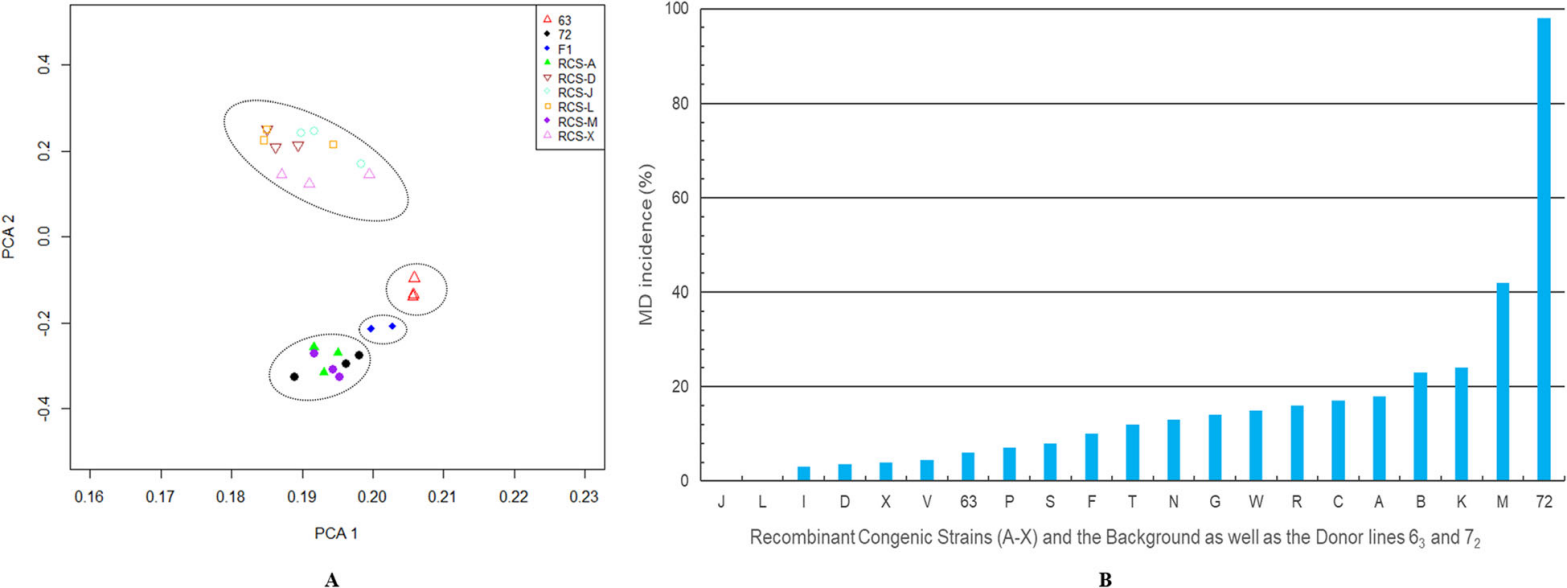
**a** PCA plot based on the first two principal components in all the nine chicken lines. These nine lines were clustered to four approximate groups, as indicated by dashed circles, which were consistent with their susceptibility to MD. **b** Histogram plot of MD incidence (%) rate. MD resistance in chickens is generally evaluated with MD incidence (induced gross tumors by MDV) and survival days post MDV challenge. The calculation was done as the ratio of number of birds with tumors/total number of birds challenged within each of the lines multiplied by 100 (cited from Xie et al. [27])

To investigate genetic structure in nine inbred chicken lines, we performed a principal component analysis (PCA) using the CNV segments by custom R scripts. Nine principal components were calculated and the first two PCs were used in the plot (Fig. 5a). The nine lines were clustered to four approximate groups with the similar patterns, as indicated by dashed circles (Fig. 5a), which were consistent with their susceptibility to MD (Fig. 5b [27]). Lines RCS-A, M and 7_2_ were well clustered together with high MD susceptibility. Lines RCS-D, J, L and X were clustered together with high resistance to MD. Interestingly enough, as expected, F_1_ individuals with a medium MD resistance were in an intermediary position between line 7_2_ and line 6_3_, which provided the theoretical basis to identify imprinting genes for disease resistance.

### Shared versus line-specific CNVRs

To investigate how frequently CNVRs were shared or lineage-specific across different lines, we calculated the CNVR numbers among the nine inbred chicken lines (Table 1). In total, 90 CNVRs were detected across all the individuals, which represented the commonly shared CNVRs. A total of 55, 44, 82, 15, 14, 72, 190, 18, and 5 CNVRs were detected as line-specific CNVRs in line 6_3_, 7_2_, F_1_, RCS-A, D, J, L, M, and X, respectively, as compared to other lines (Table 1, Additional file 6: Table S5). Importantly, the line 6_3_ and 7_2_ lineage-specific CNVRs could potentially offer certain clues to explore the genetic mechanisms of MD resistance or susceptibility. So, a total of 62 and 57 harbored genes were identified in line 6_3_ and 7_2_, respectively, including several immune-, tumor- and disease-related genes, such as *interferon regulatory factor 2* (*IRF2*), suggesting that the CNV in the *IRF2* gene is specific to line 7_2_ in this study (Additional file 7: Table S6). Interestingly, our lab also found a MD-resistance associated differentially methylated region (DMR, chr4: 38,999,001-39,000,000), which was hypermethylated in line 6_3_ compared with line 7_2_, in our previous DNA methylation study. The harbored region also included *IRF2*, which is involved in immune response IFN alpha/beta signaling pathway. This gene could be a candidate gene associated with MD susceptibility.

### CNVRs validation

**Fig. 6:**
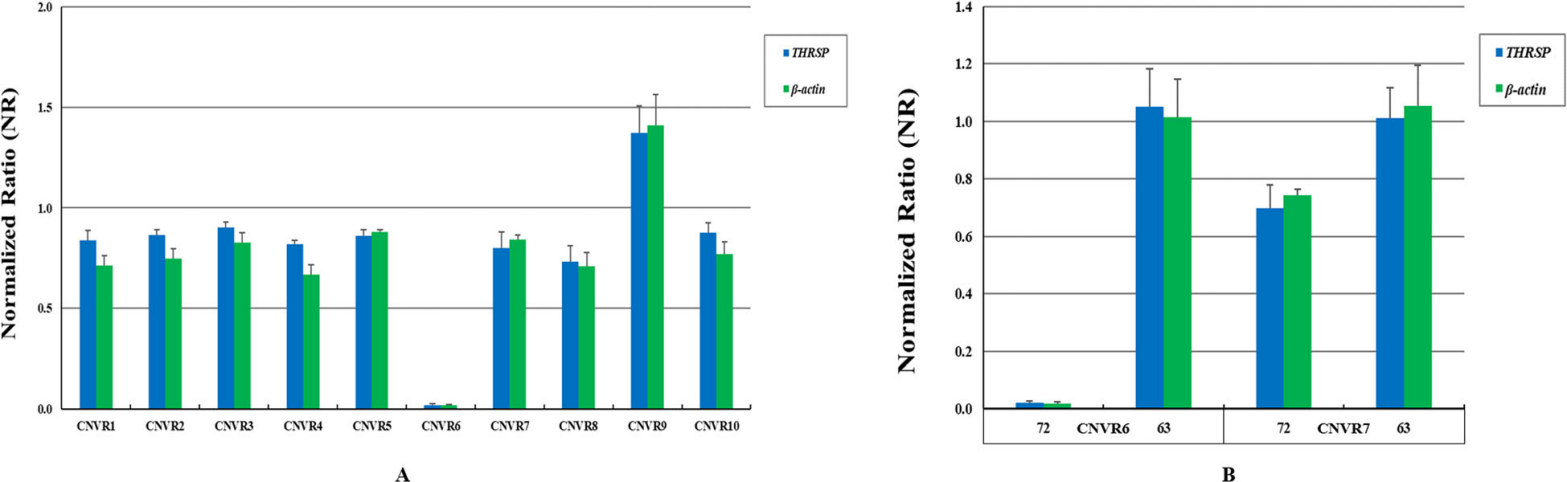
qPCR validation. **a** Normalized ratio (NR) obtained by qPCR for 10 CNVRs. The y-axis shows the NR values, and the x-axis shows the CNVR ID. **b** qPCR validation on two line 7_2_ lineage-specific deletion CNVRs. The y-axis shows the NR values, and the x-axis shows the CNVR ID and chicken lines. *THRSP* and *β-actin* served as reference genes with no variation. Samples with NR value of approximately 1 denote normal status, samples with NR value of less than 1 denote copy-number-loss status, and samples with value of about 1.5 or more denote copy-number-gain status

To confirm the identified CNVRs, 10 CNVRs containing gains (duplications) and losses (deletions) detected here were validated by qPCR using two reference genes (*THRSP* and *β-actin*). We found that all of the 10 CNVRs were confirmed in agreement with the CNVnator results (Fig. 6a), further supporting the reliability of the detected CNVRs. We also performed a qPCR validation on two line 7_2_ lineage-specific deletion CNVRs: CNVR6 (chr4: 38,999,001-39,000,200, harbored gene: *IRF2*) and CNVR7 (chr4: 82,407,001-82,409,800, harbored gene: *MAX dimerization protein 4*, *MXD4*). For CNVR6, a total deletion was detected in line 7_2_, while line 6_3_ had a normal status. For CNVR7, line 7_2_ had two third of the normal copy numbers, while line 6_3_ also had a normal status (Fig. 6b). Therefore, the copy numbers of these two loci were found significantly lower in line 7_2_ as compared to line 6_3_, again supporting our CNV calls and suggesting that they are potentially linked to MD susceptibility.

### Comparison with other studies on CNV in chickens

Considering that most of the previous CNV detection studies were based on the galGal2 and galGal3 genome assembly, coordinates of the CNVRs were converted using the UCSC liftOver tool (http://genome.ucsc.edu/cgi-bin/hgLiftOver). We migrated all chromosomal CNVRs from galGal2 and galGal3 (used in previous studies) to galGal4. We eventually obtained 585 CNVRs in the present study for comparison. Our results were then compared to 12 previous reports on chicken genomic CNV (Table 3). As a result, about 3.7, 5.2 and 4.1% of the Crooijmans et al.’s [34], Tian et al.’s [35] and Yi et al.’s [25] results can be validated in our study, respectively. Moreover, about 12.1, 4.0 and 8.8% of the Luo et al.’s [20], Yan et al.’s [21] and Xu et al.’s [22] results that also involved in MD were validated in our study. Taken together, 42.2% of our CNVRs overlapped with these three MD studies. The detailed information of CNVRs identified in this study and previous studies is provided in Additional file 8: Table S7.

**Table 3:** A summary of the chicken CNVRs identified in this study and previous studies

Study	Platform	Number of CNVRs	Total length (Mb)	Concordant number with our study
Griffin et al., 2008 [28]	NimbleGen 385 K	20	2.84	1
Skinner et al., 2009 [29]	NimbleGen 385 K	15	2.91	2
Wang et al., 2010 [30]	NimbleGen 385 K	96	16.14	4
Volker et al., 2010 [31]	NimbleGen 385 K	25	5.29	1
Wang et al., 2012 [32, 33]	Agilent 400 K	130	3.34	4
Crooijmans et al., 2013 [34]	Aglilent 244 K	1553	61.67	57
Luo et al., 2013 [20]	Agilent 400 K	33	1.92	4
Tian et al., 2013 [35]	Agilent 400 K	308	10.81	16
Zhou et al., 2014 [15]	Illumina 60 K	137	27.32	7
Yi et al., 2014 [25]	Illumina HiSeq 2000	7530	88.12	306
Yan et al., 2015 [21]	Illumina HiSeq 2000	5680	28.94	228
Xu et al., 2017 [22]	Affymetix 600 K	170	0.83	15
This study	Illumina HiSeq 2000	585	88.16	–

## Discussion

MD, a complicated tumor disease, has been used as a model for human tumor study [36]. The genetic mechanism underlying MD is likely to be very complex and remains incompletely understood. Thus, it is important to understand the genetic basis of MD-resistance or MD-susceptibility poultry, which can provide crucial clues for human diseases. In the present study, based on the high throughput sequencing platform, some bioinformatics analyses were conducted to identify CNVs, genes and enriched pathways, taking full advantage of identical genetic background in nine inbred chicken lines.

Copy number variations in the chicken genome have been explored by many research groups in the past decade. However, most of the previous studies focused on CNV discoveries using low-density SNP arrays [30, 31]. With the development of high-throughput genotyping technology, the NGS data have been used to detect the complex diseases and traits-related CNVs. CNV detections based on NGS data, which has much higher density compared to SNP chip data, have been developed and implemented in different tools [37]. There are four main methods for detecting CNVs with NGS data: Read-Pair (RP), Split Read (SR), Read Depth (RD), and assembly (AS) based methods, including CNVnator [26] used here, Pindel [38], ReadDepth [39], PEMer [40], and some other useful methods. However, each of the methods have different advantages and limitations in their applicability and suitability for NGS data. CNVnator based on RD method was the only software employed in this study. It uses the established mean-shift approach with additional corrections for multiple-bandwidth partitioning and GC correction, for more accurate CNV detection. Previous approaches using RD were limited to only unique regions of the genome and discovered only large CNVs with poor breakpoint resolution, or could not perform genotyping. CNVnator is able to discover CNVs in a vast range of sizes, from a few hundred bp to several Mb in length, in the whole genome. Therefore, our results here could reveal additional novel genetic variations underlying MD than those revealed by SNP arrays alone.

In the present study, we performed comparisons with the previous CNV studies, especially three researches also involved in MD. We found 247 CNVRs covering 93.9 Mb in length that overlapped with these three MD studies. It is interesting to note that only 7 (1.2%) and 15 (2.6%) CNVRs were shared with Luo et al.’s [20] and Xu et al.’s [22] results using SNP chip data, which may be, in part, related to limited sample sizes, different platforms, different analysis methods, and different chicken genome references (although we converted the genome positions from two previous genome assemblies (galGal2 and galGal3) to a newer one (galGal4) with the help of LiftOver based on UCSC, some information may still be missing). More importantly, we also used different chicken lines, especially the selected RCSs from a total of 19 RCSs (Fig. 1). We also compared with the CNV identified in lines 6_3_ and 7_2_ using NGS data for MD study [21], and found that 228 (39.0%) CNVRs overlapped, which provided more effective information for our study. Moreover, our study explored the genetic structure based on CNV in different inbred chicken lines. The PCA showed clearly that the first two PCs can divide all chickens into four unique groups, which is similar to the results of Xu et al. [22]. Therefore, our study further confirms that CNV markers can be used to study the genetic variability in diverse chicken lines, which could possibly contribute to lineage-specific phenotypes.

The genetic mechanism underlying MD is likely to be very complex and is not clear yet. It may be determined by some specific structural variations but not a single gene or a SNP mutation, though several candidate genes have been reported in previous studies described above. In the current study, we investigated CNVs among diverse inbred lines and found 55 and 44 unique CNVRs in lines 6_3_ and 7_2_, respectively, which could be associated with MD. Notably, we successfully identified a CNVR, which was a deletion and a normal copy number in all individuals from line 7_2_ and line 6_3_, respectively, including a nearby gene *IRF2*. Fortunately, the *IRF2* gene was also highlighted in our previous DNA methylation study involved in a critical DMR identified by methyl-CpG binding domain protein enriched genome sequencing (MBD-seq) with a false discovery rate (FDR) < 0.1 and validated by bisulfite cloning sequencing, which was hypermethylated in line 6_3_ compared with line 7_2_. The region of the DMR identified in previous study and the CNVR identified here was almost completely the same, with the same start site and a 1000 bp overlaps. The nearby gene *IRF2* is a disease- and virus-related gene involved in the interferon gamma signaling pathway and the immune response IFN alpha/beta signaling pathway [41]. This gene is conserved in human and some other species like chimpanzee, Rhesus monkey, dog, cow, mouse, rat, zebrafish, and frog. Thus, some mutations or structural variations of this gene could be key factors related to disorders or diseases. It was reported that *IRF2* gene was associated with several diseases in chickens like necrotic enteritis [42], pancreatic cancer [43], and atopic dermatitis and eczema herpeticum [44]. More interestingly, this gene can specifically bind to the upstream regulatory region of type I IFN and IFN-inducible MHC class I genes, which could be an important clue to explore the genetic mechanisms of MD resistance because, to our knowledge, MHC plays an important role in the determination of resistance to MD [1]. Therefore, *IRF2* may be a very important gene related to MD according to the structural variation identified here, its known functions and our former studies. Another useful information obtained in this study is the JAK/STAT signaling pathway, which was also considered as a potential pathway responding to MDV infection reported by Perumbakkam et al. [45]. The JAK/STAT signaling pathway is one of a handful of pleiotropic cascades used to transduce a multitude of signals for development and homeostasis in animals [46]. JAK activation stimulates cell proliferation, differentiation, cell migration and apoptosis. These cellular events are critical to immune development and some other processes. Importantly, mutations that constitutively activate or fail to regulate JAK signaling properly cause inflammatory disease, including several chicken diseases [47, 48]. Additionally, a previous study reported that *IRF2* can regulate macrophage apoptosis through a STAT1/3 [49], which provides valuable and potential interaction of *IRF2* and JAK/STAT pathway and might jointly contribute to the genetic resistance to MD. Therefore, we hypothesize a probable mechanism of complex disease: the deletions in CNV could be associated with different epigenetic effects, which further regulate an interacting pathway leading to occurrence of diseases.

## Conclusions

In summary, we investigated copy number variations in inbred chicken lines using next generation sequencing. We have successfully identified a number of line-specific CNVRs, as well as revealed genes and pathways that may be involved in genetic resistance to MD. Combined with our previous study and due to the complexity of MD, we ultimately found a high-confidence candidate gene *IRF2*, and an immune- or disease-related pathway, JAK/STAT signaling pathway, which could jointly play potentially important roles in response to MD resistance. Overall, our findings in the present study will provide valuable insights for understanding the genetic mechanism of resistance to MD and will be worthy of further functional characterization.

## Methods

### Experimental population

A total of 26 female chickens without treatments were used for blood collection in this study, including three chickens from each of the line 6_3_ (MD-resistant), line 7_2_ (MD-susceptible) and six recombinant congenic strains (RCSs, RCS-A, D, J, L, M, and X), and two chickens from reciprocal cross F_1_ hybrid 6_3_ × 7_2_ (USDA-ARS ADOL, East Lansing, Michigan, USA) [50]. RCSs were developed using line 6_3_ as the parental strain mated to line 7_2_ and then backcrossed to line 6_3_ twice followed by full-sib mating for more than 20 generations (Fig. 1). Eventually, diverse RCSs were generated and they contain 87.5% of line 6_3_ and 12.5% of line 7_2_ in the genetic background but differ in MD resistance/susceptibility [51]. All the experimental chickens were anesthetized with sodium pentobarbital (intraperitoneal injection: 150 mg/kg) at the end of this study.

### Library construction and sequencing

Blood samples were collected from the brachial vein by venipuncture. Genomic DNA (gDNA) from blood samples was extracted using the DNeasy Blood & Tissue Mini Kit (Qiagen, USA) according to the manufacturer’s instructions. The purity and concentration of the gDNA samples were measured by NanoDrop ND-1000 spectrophotometer (Thermo Scientific, USA) and by agarose gel electrophoresis. After the examinations, paired-end libraries were generated for each eligible sample using standard procedures (Illumina, USA). The average insert size was 500 bp, and the average read length was 100 bp for line 6_3_ and line 7_2_, and 150 bp for the remaining chicken lines. All libraries were sequenced on an Illumina® HiSeq 2000 sequencing platform to an average raw read sequence coverage of × 20 for lines 6_3_, 7_2_ and their F_1_ hybrid, and × 6 for the six RCSs, respectively. The depth ensured the accuracy and reduced the false-negative rate of CNV calling for downstream analysis. Library preparation and all Illumina runs were performed as the standard manufacturer’s protocols.

### Read alignment and CNV calling

Chicken genome assembly (galGal4) was retrieved from the UCSC Genome Browser website (http://hgdownload.soe.ucsc.edu/goldenPath/galGal4/bigZips/) [52]. In order to minimize the mapping errors, quality control was performed by FastQC [53] and low quality reads were removed with the help of FastX Toolkit (Gordon A and Hannon G: Fastx-toolkit: FASTQ/A short-reads preprocessing tools, unpublished) and Trimmomatic using the default parameters. The resulting FastQ files of mapping reads of each sample were aligned to the reference genome individually using Burrows-Wheeler Aligner (BWA-MEM) (v0.7.15) [54] with mainly default parameters. SAMtools (v1.3) [55] was then used to convert the alignment results (SAM format) to BAM format and all converted BAM files were sorted with the command SAMtools. Duplicate reads were removed from individual sample alignments using MarkDuplicates in the Picard package (http://broadinstitute.github.io/picard/) to avoid any influence on variant detection, and reads were merged using MergeSam-Files. We additionally performed local realignment using Genome Analysis Toolkit (GATK, v3.5) [56] to enhance the alignments in regions of indel polymorphisms, which can greatly improve the sensitivity and specificity in CNV calling [57].

After mapping, CNV calling was performed using CNVnator (v0.3.3) software [26] based on read depth (RD) method to predict genomic CNVs between the nine chicken lines and the reference. CNVnator firstly calculated the counts of mapped reads within user specified non-overlapping bins of equal size as the RD signal, and then adjusted the signal in consideration of the potential correlation between RD signal and GC content of the underlying genomic sequence. The mean-shift algorithm was employed to segment the signal with presumably different underlying CN. Then CNVs were predicted by applying statistical significance tests to the segments. We then ran CNVnator with a bin size of 100 bp for our data. CNV calls were filtered using stringent criteria including *P*-value < 0.05 and minimum size > 1 Kb, and calls with > 50% of q0 (zero mapping quality) reads within the CNV regions were removed (q0 filter). All CNV calls overlapping with gaps in the reference genome were excluded from consideration. CNVs located on random contigs (chrN_random), unlocalized chromosomes (chrUn), or in overlapping gaps were discarded for further analysis due to the shorter length of the chrUn contigs and mapping ambiguity of chrUn sequence reads. In order to compare our results with previous studies, we converted all chromosomal CNVRs from galGal2 and galGal3 (used in previous studies) to galGal4 with the assistance of LiftOver based on UCSC (http://genome.ucsc.edu/cgi-bin/hgLiftOver).

### Gene detection and functional analysis

Results from CNVnator were combined to obtain a collective set of unique CNVs with different start or end coordinates. These CNVs were then merged into non-overlapping CNV regions (CNVRs) by aggregating CNVs that overlap by at least 1 bp across all samples of each chicken line. The Ensembl genes (release 85 Database) were obtained using BioMart software based on the chicken gene sequence assembly (galGal4) and the genes harbored in the inferred CNVRs were extracted using custom Perl scripts. The Database for Annotation, Visualization, and Integrated Discovery (DAVID, version 6.8) (https://david.ncifcrf.gov/) [58] was used to perform the gene ontology (GO) enrichment analysis and Kyoto Encyclopedia of Genes and Genomes (KEGG) pathway analysis.

### Validation of CNVRs by quantitative real-time PCR (qPCR)

To experimentally validate the detected CNV calls by CNVnator, we performed qPCR confirmation of ten CNVRs randomly selected from line 6_3_ and line 7_2_, respectively, using gDNA samples from different chicken lines. All the primers were designed based on the GenBank reference sequences using the Primer 3.0 webtool (http://frodo.wi.mit.edu/primer3/) (Additional file 9: Table S8). The *β-*actin gene and thyroid hormone responsive (*THRSP*) gene served as reference genes. For each chicken line, at least three individuals were used to do the validation. qPCR using SYBR Green PCR Kit was performed in triplicate based on iCycler iQ PCR System (Bio-Rad). qPCR program was run as follows: pre-incubation (95 °C for 10 min), 40 cycles of amplification (95 °C for 10 s, 60 °C for 10 s, and 72 °C for 10 s), melting curves using a heat ramp and cool down. Cycle threshold values (Ct values) were obtained from iCycler iQ PCR software. The 2^-ΔΔCT^ method was used to calculate the copy number [59–61]. The corresponding equation was:

$$ \varDelta \varDelta CT={\left({CT}_{target\ gene}-{CT}_{reference\ gene}\right)}_{sample\ A}-{\left({CT}_{target\ gene}-{CT}_{reference\ g\mathrm{e} ne}\right)}_{sample\ B}, $$

where CT is the threshold cycle, sample A is the tested individual, and sample B is the control individual with single copy or no variation in copy number. Samples with Normal Ratio (NR) about 1 denote normal individuals (two copies), samples with NR of less than 1 denote one copy loss individuals, and samples with NR about 1.5 or more denote copy number gain individuals [32, 33].

## Supplementary information

**Additional file 1: Table S1.** Summary statistics for sequencing.

**Additional file 2: Table S2.** Summary statistics of raw and screened CNVs.

**Additional file 3: Table S3.** The detailed lists of clusters with enrichment scores > 1.0 from DAVID.

**Additional file 4: Figure S1.** JAK/STAT signaling pathway.

**Additional file 5: Table S4.** The detailed lists of all the GO terms and pathways from DAVID.

**Additional file 6: Table S5.** Lists of line-specific and shared CNVRs in all the chicken lines.

**Additional file 7: Table S6.** Lists of disease-related genes in line 6_3_ and line 7_2_, respectively.

**Additional file 8: Table S7.** The detailed information of CNVRs identified in this study and previous studies.

**Additional file 9: Table S8.** All the primer sequences used in qPCR for CNVRs validation.

## Data Availability

The sequencing data have been submitted to the NCBI Sequence Read Archive (SRA), and are accessible through the accession number PRJNA641254.

## Abbreviations

ADOL: Avian disease and oncology laboratory
CGH: Comparative genomic hybridization;
chr: Chromosome
CNV: Copy number variation
CNVR: Copy number variation region
DAVID: The database for annotation visualization and integrated discovery
DMR: Differentially methylated region
dpi: Days post infection
FDR: False discovery rate
gDNA: Genomic DNA
GO: Gene ontology
*IRF2*: Interferon regulatory factor 2
Kb: Kilobases
KEGG: Kyoto encyclopedia of genes and genomes
Mb: Megabases
MBD-seq: Methyl-CpG binding domain protein enriched genome sequencing
MD: Marek’s disease
MDV: Marek’s disease virus
*MXD4*: MAX dimerization protein 4
NGS: Next generation sequencing
PCA: Principal component analysis
RCS: Recombinant congenic strain
*THRSP*: Thyroid hormone responsive

## References

[1] Bacon L, Hunt H, Cheng H (2000). A review of the development of chicken lines to resolve genes determining resistance to diseases. Poult Sci.

[2] Calnek B, Witter RL (1985). Marek's disease-a model for herpesvirus oncology. CRC Crit Rev Microbiol.

[3] Calnek B. Pathogenesis of Marek's disease virus infection. In: Marek's Disease. Berlin: Springer; 2001:25–55. 10.1007/978-3-642-56863-3_2 11217426

[4] Weischenfeldt J, Symmons O, Spitz F, Korbel JO (2013). Phenotypic impact of genomic structural variation: insights from and for human disease. Nat Rev Genet.

[5] Redon R, Ishikawa S, Fitch KR, Feuk L, Perry GH, Andrews TD, Fiegler H, Shapero MH, Carson AR, Chen W (2006). Global variation in copy number in the human genome. Nature.

[6] Zhang F, Gu W, Hurles ME, Lupski JR (2009). Copy number variation in human health, disease, and evolution. Annu Rev Genomics Hum Genet.

[7] Iafrate AJ, Feuk L, Rivera MN, Listewnik ML, Donahoe PK, Qi Y, Scherer SW, Lee C (2004). Detection of large-scale variation in the human genome. Nat Genet.

[8] Sebat J, Lakshmi B, Troge J, Alexander J, Young J, Lundin P, Månér S, Massa H, Walker M, Chi M (2004). Large-scale copy number polymorphism in the human genome. Science.

[9] Fiegler H, Redon R, Andrews D, Scott C, Andrews R, Carder C, Clark R, Dovey O, Ellis P, Feuk L (2006). Accurate and reliable high-throughput detection of copy number variation in the human genome. Genome Res.

[10] Glessner JT, Wang K, Cai G, Korvatska O, Kim CE, Wood S, Zhang H, Estes A, Brune CW, Bradfield JP (2009). Autism genome-wide copy number variation reveals ubiquitin and neuronal genes. Nature.

[11] Conrad DF, Pinto D, Redon R, Feuk L, Gokcumen O, Zhang Y, Aerts J, Andrews TD, Barnes C, Campbell P (2010). Origins and functional impact of copy number variation in the human genome. Nature.

[12] Fontanesi L, Beretti F, Riggio V, Gómez González E, Dall’Olio S, Davoli R, Russo V, Portolano B (2009). Copy number variation and missense mutations of the agouti signaling protein (ASIP) gene in goat breeds with different coat colors. Cytogenet Genome Res.

[13] Wright D, Boije H, Meadows JR, Bed'Hom B, Gourichon D, Vieaud A, Tixier-Boichard M, Rubin C-J, Imsland F, Hallböök F (2009). Copy number variation in intron 1 of SOX5 causes the pea-comb phenotype in chickens. PLoS Genet.

[14] Bickhart DM, Hou Y, Schroeder SG, Alkan C, Cardone MF, Matukumalli LK, Song J, Schnabel RD, Ventura M, Taylor JF (2012). Copy number variation of individual cattle genomes using next-generation sequencing. Genome Res.

[15] Zhou W, Liu R, Zhang J, Zheng M, Li P, Chang G, Wen J, Zhao G (2014). A genome-wide detection of copy number variation using SNP genotyping arrays in Beijing-you chickens. Genetica.

[16] Yi G, Qu L, Chen S, Xu G, Yang N (2015). Genome-wide copy number profiling using high-density SNP array in chickens. Anim Genet.

[17] Wu Y, Fan H, Jing S, Xia J, Chen Y, Zhang L, Gao X, Li J, Gao H, Ren H (2015). A genome-wide scan for copy number variations using high-density single nucleotide polymorphism array in Simmental cattle. Anim Genet.

[18] Zhu C, Fan H, Yuan Z, Hu S, Ma X, Xuan J, Wang H, Zhang L, Wei C, Zhang Q. Genome-wide detection of CNVs in Chinese indigenous sheep with different types of tails using ovine high-density 600K SNP arrays. Sci Rep. 2016;6:27822. 10.1038/srep27822 PMC4901276 27282145

[19] Bai H, Sun Y, Liu N, Liu Y, Xue F, Li Y, Xu S, Ni A, Ye J, Chen Y (2018). Genome-wide detection of CNV s associated with beak deformity in chickens using high-density 600K SNP arrays. Anim Genet.

[20] Luo J, Yu Y, Mitra A, Chang S, Zhang H, Liu G, Yang N, Song J (2013). Genome-wide copy number variant analysis in inbred chickens lines with different susceptibility to Marek’s disease. G3: genes. Genomes, Genet.

[21] Yan Y, Yang N, Cheng HH, Song J, Qu L (2015). Genome-wide identification of copy number variations between two chicken lines that differ in genetic resistance to Marek’s disease. BMC Genomics.

[22] Xu L, He Y, Ding Y, Sun G, Carrillo J, Li Y, Ghaly M, Ma L, Zhang H, Liu G (2017). Characterization of copy number variation’s potential role in Marek’s disease. Int J Mol Sci.

[23] Zhan B, Fadista J, Thomsen B, Hedegaard J, Panitz F, Bendixen C (2011). Global assessment of genomic variation in cattle by genome resequencing and high-throughput genotyping. BMC Genomics.

[24] Clop A, Vidal O, Amills M (2012). Copy number variation in the genomes of domestic animals. Anim Genet.

[25] Yi G, Qu L, Liu J, Yan Y, Xu G, Yang N (2014). Genome-wide patterns of copy number variation in the diversified chicken genomes using next-generation sequencing. BMC Genomics.

[26] Abyzov A, Urban AE, Snyder M, Gerstein M (2011). CNVnator: an approach to discover, genotype, and characterize typical and atypical CNVs from family and population genome sequencing. Genome Res.

[27] Xie Q, Chang S, Dong K, Dunn JR, Song J, Zhang H (2017). Genomic variation between genetic lines of white leghorns differed in resistance to marek's disease. J Clin Epigenetics.

[28] Griffin DK, Robertson LB, Tempest HG, Vignal A, Fillon V, Crooijmans RP, Groenen MA, Deryusheva S, Gaginskaya E, Carré W. Whole genome comparative studies between chicken and turkey and their implications for avian genome evolution. BMC Genomics. 2008;9:168. 10.1186/1471-2164-9-168 PMC2375447 18410676

[29] Skinner BM, Robertson LB, Tempest HG, Langley EJ, Ioannou D, Fowler KE, Crooijmans RP, Hall AD, Griffin DK, Völker M (2009). Comparative genomics in chicken and Pekin duck using FISH mapping and microarray analysis. BMC genomics..

[30] Wang X, Nahashon S, Feaster TK, Bohannon-Stewart A, Adefope N (2010). An initial map of chromosomal segmental copy number variations in the chicken. BMC Genomics.

[31] Völker M, Backström N, Skinner BM, Langley EJ, Bunzey SK, Ellegren H, Griffin DK (2010). Copy number variation, chromosome rearrangement, and their association with recombination during avian evolution. Genome Res.

[32] Wang J, Jiang J, Fu W, Jiang L, Ding X, Liu J-F, Zhang Q (2012). A genome-wide detection of copy number variations using SNP genotyping arrays in swine. BMC Genomics.

[33] Wang Y, Gu X, Feng C, Song C, Hu X, Li N (2012). A genome-wide survey of copy number variation regions in various chicken breeds by array comparative genomic hybridization method. Anim Genet.

[34] Crooijmans RP, Fife MS, Fitzgerald TW, Strickland S, Cheng HH, Kaiser P, Redon R, Groenen MA (2013). Large scale variation in DNA copy number in chicken breeds. BMC Genomics.

[35] Tian M, Wang Y, Gu X, Feng C, Fang S, Hu X, Li N (2013). Copy number variants in locally raised Chinese chicken genomes determined using array comparative genomic hybridization. BMC Genomics.

[36] Osterrieder N, Kamil JP, Schumacher D, Tischer BK, Trapp S (2006). Marek's disease virus: from miasma to model. Nat Rev Microbiol.

[37] Pirooznia M, Goes FS, Zandi PP (2015). Whole-genome CNV analysis: advances in computational approaches. Front Genet.

[38] Ye K, Schulz MH, Long Q, Apweiler R, Ning Z (2009). Pindel: a pattern growth approach to detect break points of large deletions and medium sized insertions from paired-end short reads. Bioinformatics.

[39] Miller CA, Hampton O, Coarfa C, Milosavljevic A (2011). ReadDepth: a parallel R package for detecting copy number alterations from short sequencing reads. PLoS One.

[40] Korbel JO, Abyzov A, Mu XJ, Carriero N, Cayting P, Zhang Z, Snyder M, Gerstein MB (2009). PEMer: a computational framework with simulation-based error models for inferring genomic structural variants from massive paired-end sequencing data. Genome Biol.

[41] Mizutani T, Tsuji K, Ebihara Y, Taki S, Ohba Y, Taniguchi T, Honda K (2008). Homeostatic erythropoiesis by the transcription factor IRF2 through attenuation of type I interferon signaling. Exp Hematol.

[42] Broom LJ, Kogut MH (2018). Deciphering desirable immune responses from disease models with resistant and susceptible chickens. Poult Sci.

[43] Cui L, Deng Y, Rong Y, Lou W, Mao Z, Feng Y, Xie D, Jin D (2012). IRF-2 is over-expressed in pancreatic cancer and promotes the growth of pancreatic cancer cells. Tumor Biol.

[44] Gao P-S, Leung DY, Rafaels NM, Boguniewicz M, Hand T, Gao L, Hata TR, Schneider LC, Hanifin JM, Beaty TH (2012). Genetic variants in interferon regulatory factor 2 (IRF2) are associated with atopic dermatitis and eczema herpeticum. J Investig Dermatol.

[45] Perumbakkam S, Muir WM, Black-Pyrkosz A, Okimoto R, Cheng HH (2013). Comparison and contrast of genes and biological pathways responding to Marek’s disease virus infection using allele-specific expression and differential expression in broiler and layer chickens. BMC Genomics.

[46] Rawlings JS, Rosler KM, Harrison DA (2004). The JAK/STAT signaling pathway. J Cell Sci.

[47] Sandford EE, Orr M, Balfanz E, Bowerman N, Li X, Zhou H, Johnson TJ, Kariyawasam S, Liu P, Nolan LK (2011). Spleen transcriptome response to infection with avian pathogenic Escherichia coli in broiler chickens. BMC Genomics.

[48] Lin J, Xia J, Zhang K, Yang Q (2016). Genome-wide profiling of chicken dendritic cell response to infectious bursal disease. BMC Genomics.

[49] Cuesta N, Nhu QM, Zudaire E, Polumuri S, Cuttitta F, Vogel SN (2007). IFN regulatory factor-2 regulates macrophage apoptosis through a STAT1/3-and caspase-1-dependent mechanism. J Immunol.

[50] Stone HA. Use of highly inbred chickens in research. Washington DC: USDA Agriculture Research Service Technical Bulletin; 1975.

[51] Chang S, Ding Z, Dunn JR, Lee LF, Heidari M, Song J, Ernst CW, Zhang H (2011). A comparative evaluation of the protective efficacy of rMd5ΔMeq and CVI988/Rispens against a vv+ strain of Marek's disease virus infection in a series of recombinant congenic strains of white Leghorn chickens. Avian Dis.

[52] Meyer LR, Zweig AS, Hinrichs AS, Karolchik D, Kuhn RM, Wong M, Sloan CA, Rosenbloom KR, Roe G, Rhead B (2013). The UCSC genome browser database: extensions and updates 2013. Nucleic Acids Res.

[53] Schmieder R, Edwards R (2011). Quality control and preprocessing of metagenomic datasets. Bioinformatics.

[54] Bolger AM, Lohse M, Usadel B. Trimmomatic: a flexible trimmer for Illumina sequence data. Bioinformatics 2014:30(15):2114–2120. 10.1093/bioinformatics/btu170 PMC4103590 24695404

[55] Li H, Handsaker B, Wysoker A, Fennell T, Ruan J, Homer N, Marth G, Abecasis G, Durbin R (2009). The sequence alignment/map format and SAMtools. Bioinformatics.

[56] McKenna A, Hanna M, Banks E, Sivachenko A, Cibulskis K, Kernytsky A, Garimella K, Altshuler D, Gabriel S, Daly M (2010). The genome analysis toolkit: a MapReduce framework for analyzing next-generation DNA sequencing data. Genome Res.

[57] DePristo MA, Banks E, Poplin R, Garimella KV, Maguire JR, Hartl C, Philippakis AA, Del Angel G, Rivas MA, Hanna M (2011). A framework for variation discovery and genotyping using next-generation DNA sequencing data. Nat Genet.

[58] Huang DW, Sherman BT, Lempicki RA (2009). Systematic and integrative analysis of large gene lists using DAVID bioinformatics resources. Nat Protoc.

[59] Livak KJ, Schmittgen TD (2001). Analysis of relative gene expression data using real-time quantitative PCR and the 2-ΔΔCT method. Methods.

[60] Bodin L, Beaune PH, Loriot M-A (2005). Determination of cytochrome P450 2D6 (CYP2D6) gene copy number by real-time quantitative PCR. Biomed Res Int.

[61] D’haene B, Vandesompele J, Hellemans J (2010). Accurate and objective copy number profiling using real-time quantitative PCR. Methods.

