# Supplementary figures and images for: Genome-wide characterization of copy number variations in the host genome in genetic resistance to Marek’s disease using next generation sequencing
Source: BMC Genet. 2020 Jul 16;21:77. doi: 10.1186/s12863-020-00884-w (PMC7364486; doi:10.1186/s12863-020-00884-w)

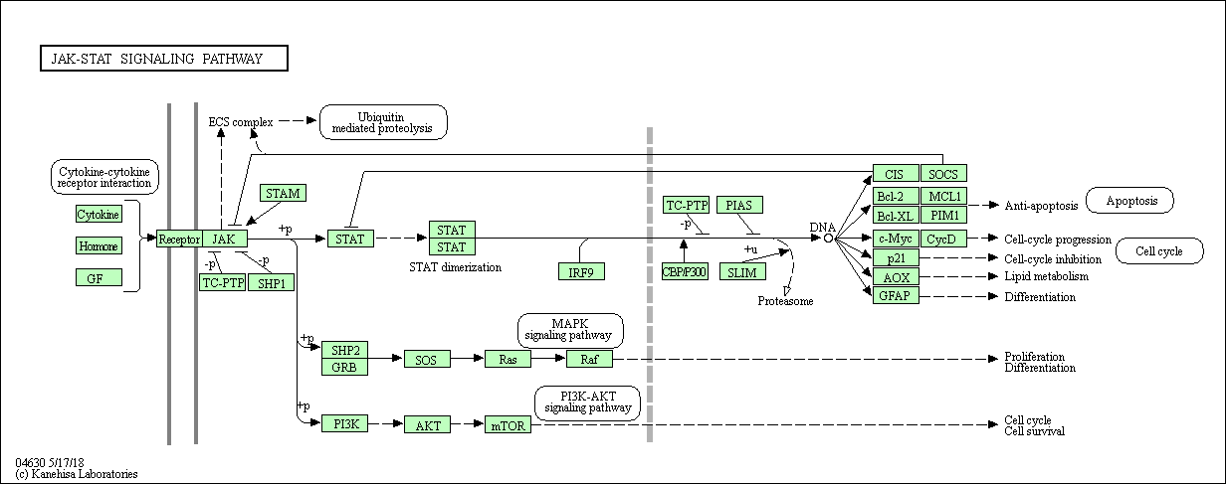

Supplement: Supplementary file 4 — Additional file 4: Figure S1. JAK/STAT signaling pathway. [file 12863_2020_884_MOESM4_ESM.tif]
